# Triphenylmethane Derivatives Have High *In Vitro* and *In Vivo* Activity against the Main Causative Agents of Cutaneous Leishmaniasis

**DOI:** 10.1371/journal.pone.0051864

**Published:** 2013-01-14

**Authors:** Renata Celi Carvalho de Souza Pietra, Lucas Fonseca Rodrigues, Eliane Teixeira, Levi Fried, Benjamin Lefkove, Ana Rabello, Jack Arbiser, Lucas Antônio Miranda Ferreira, Ana Paula Fernandes

**Affiliations:** 1 Department of Clinical and Toxicology Analyses, Federal University of Minas Gerais (UFMG), Belo Horizonte, Minas Gerais, Brazil; 2 Laboratory of Clinical Research, Instituto René Rachou, Fundação Oswaldo Cruz, Belo Horizonte, Minas Gerais, Brasil; 3 Department of Dermatology, Emory University School of Medicine, Atlanta, Georgia, United States of America; Universidade Estadual De Campinas, Biology Institute, Brazil

## Abstract

The current standard of care for cutaneous leishmaniasis (CL) is organic antimonial compounds, but the administration of these compounds is complicated by a low therapeutic - toxic index, as well as parenteral administration. Thus, there is an urgent need for the development of new and inexpensive therapies for the treatment of CL. In this study, we evaluate the activity of the triphenylmethane (TPM) class of compounds against three species of *Leishmania* which are pathogenic in humans. The TPM have a history of safe use in humans, dating back to the use of the original member of this class, gentian violet (GV), from the early 20^th^ century. Initially, the *in vitro* efficacy against *Leishmania (Viannia) braziliensis*, *L. (Leishmania) amazonensis* and *L. (L.) major* of 9 newly synthesized TPM, in addition to GV, was tested. Inhibitory concentrations (IC) IC_50_ of 0.025 to 0.84 µM had been found in promastigotes *in vitro* assays. The four most effective compounds were then tested in amastigote intracellular assays, resulting in IC_50_ of 0.10 to 1.59 µM. A high degree of selectivity of antiparasitic activity over toxicity to mammalian cells was observed. Afterwards, GV and TPM 6 were tested in a topical formulation in mice infected with *L. (L.) amazonensis* leading to elimination of parasite burdens at the site of lesion/infection. These results demonstrated that TPM present significant anti-leishmanial activities and provide a rationale for human clinical trials of GV and other TPM. TPM are inexpensive and safe, thus using them for treatment of CL may have a major impact on public health.

## Introduction

The genus *Leishmania spp.* protozoa are pathogenic to a wide variety of hosts, including humans, and are most prevalent in tropical climates of developing countries. The major forms of leishmaniasis include cutaneous, mucosal and visceral leishmaniasis [1]. *Leishmania (Leishmania) major* is one the main etiological agents of CL in the Old World, while *Leishmania (Viannia) braziliensis*, *Leishmania (L.) amazonensis* and *Leishmania (V.) guyanensis* are the main causative species of CL in the Americas. Lesions caused by these species frequently appear as ulcers at the site of infection and are commonly located in poorly-protected areas of the body, such as the face, arms and legs [2], [3]. In addition, *L. (L.) amazonensis* may also lead to development diffuse cutaneous leishmaniasis in a few patients, which is characterized by nodular lesions, refractory to chemotherapy [4].

Current therapeutic alternatives for CL treatment are unsatisfactory. The conventional first-line therapy consists of pentavalent antimonials (sodium stibogluconate - Pentostan™ and meglumine antimoniate - Glucantime™). However, these drugs present inconvenient aspects that limit their use, such as the necessity of parenteral administration and a high incidence of toxic and adverse reactions [2]. Pentavalent antimonials have long been considered highly effective [3], [5], however, there is a growing body of evidence of variable efficacy, depending on species, geographic region, presence of resistant strains, and therapeutic schemes [2], [6]–[8]. Among the alternative therapeutic schemes, intralesional administration of pentavalent antimonials has been used to treat old world cutaneous leishmaniasis [9].

The second line therapies for leishmaniasis include amphotericin B (AmB), liposomal AmB, and pentamidine. AmB is a very powerful polyeneic antibiotic against *Leishmania* but also presents significant adverse effects, including nephrotoxicity and infusion reactions. Liposomal AmB was developed to improve the tolerability profile of AmB deoxycholate [10]. In Brazil, liposomal AmB is recommended for CL treatment only upon failure of first line therapies. In addition, another limitation of liposomal AmB is its high cost [11]. Pentamidine is complicated by hypoglycemia and the requirement of intravenous administration.

Finally paromomycin, an aminoglycoside antibiotic, is an antileishmanial drug that has been on the market since the 1960's and has been used in several formulations for the topical treatment of CL with inconclusive results [12]–[17]. Therefore, further research and studies based on new technologies aimed at improving the delivery and efficacies of topical treatments are still required, especially in regards to safety, efficacy, and cost [18].

Compounds with the triphenylmethane pharmacophore (TPM), such as gentian violet (GV), have a long history of human use as anti-bacterial and antimycotic agents. In addition, GV has been shown to have antiparasitic activity against various human parasites and have been used in blood banks to circumvent Chaga's disease transmission [19]–[24]. However, TPM have not been previously evaluated *in vivo* against CL.

We synthesized 9 novel TPM derivatives, as part of a structure-function study of TPM compounds and tested, in addition to GV, against 3 species of pathogenic *Leishmania*, including both Old World and New World *Leishmania*. These derivatives were tested against both promastigotes of *L. (L.) amazonensis*, *L. (L.) major* and *L. (V.) braziliensis* and intracellular amastigotes of *L. (L.) amazonensis* and *L. (V.) braziliensis*. Finally, we demonstrate that topical treatment with either GV or one of the novel TPM is highly effective in treating *L. (L.) amazonensis* infected mice.

## Materials and Methods

### Ethics statement

This study has been approved by Ethics Committee for Animal Experimentation from University Federal of Minas Gerais (CETEA/UFMG: 12/2009).The University Federal of Minas Gerais adheres to the standards as outlined by relevant national (CONCEA - Brazilian Government Council for Control of Animal Experimentation) and international guidelines for care and use of laboratory animals.

### Parasites

Promastigotes of *L. (L.) amazonensis* (IFLA/BR/1967/PH-8), *L. (V.) braziliensis* (MHOM/BR/75/M2903), and *L. (L.) major* (MHOM/IL/80/Friedlin) were maintained at 23°C in Schneider's Drosophila medium (Merck, Germany) supplemented with 20% heat-inactivated fetal calf serum (FCS) (Gibco, Eggenstein, Germany), pH 7.2. The same strain of *L. (L.) amazonensis* was used for both *in vitro* and *in vivo* experiments.

### Triphenylmethane compounds (TPM)

**Figure 1:**
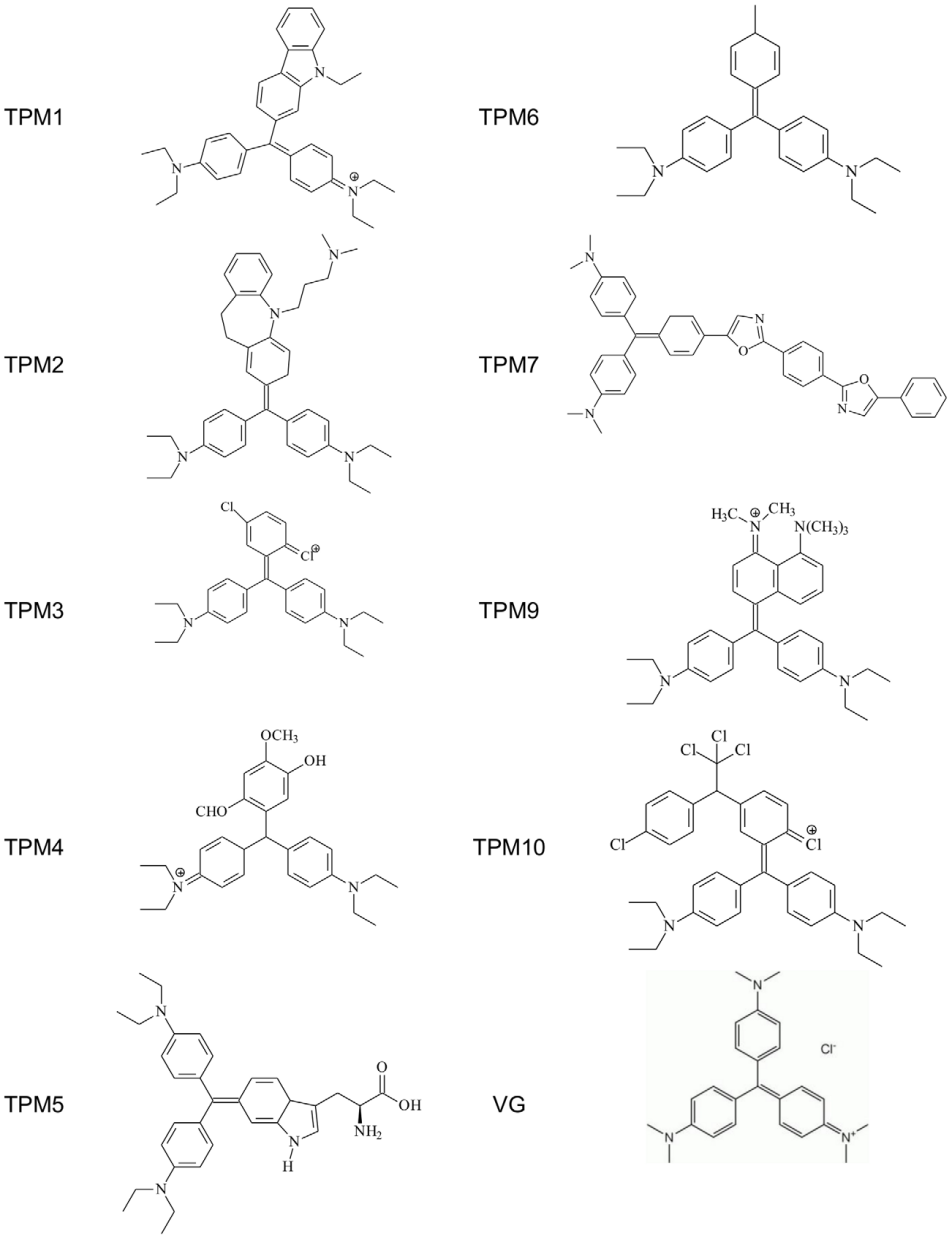
TPM structures.

Novel TPM were synthesized by reacting aromatic substrates with 4,4′bis (diethylaminobenzophenone) in the presence of phosphorus oxychloride in a calorimeter bomb at 140°C under pressure. TPM formula and molecular weight are present in table 1 and structures in figure 1. All 124 reagents were obtained from Sigma-Aldrich, and were purified on silica column 125 chromatography. The molecular weight of TPM compounds were obtained by mass spectrometry. For *in vitro* assays, a stock solution was prepared in ethanol (EtOH) and maintained at −20°C. All subsequent dilutions were prepared in the respective fresh culture Schneider's medium on the day of the assay, and the final maximum concentration of EtOH was 0.1%.

**Table 1:** Molecular weight and chemical formula for all TPM compounds tested.

TPM	Molecular Weight	Formula
TPM1	502.71	C_35_H_40_N_3_
TPM2	588.87	C_40_H_53_N_4_
TPM3	455.46	C_27_H_31_Cl_2_N_2_
TPM4	432.60	C_29_H_37_N_2_O_3_
TPM5	512.69	C_32_H_40_N_4_O_2_
TPM6	400.60	C_28_H_36_N_2_
TPM7	672.86	C_41_H_38_N_4_O_2_
TPM9	521,76	C_35_H_46_N_4_
TPM10	662.95	C_35_H_37_Cl_5_N_2_
VG	407.98	C_25_H_30_ClN_3_

### Promastigotes assay

Promastigotes of *L. (L.) amazonensis*, *L. (V.) braziliensis* or *L. (L.) major* with 3 or 4 days of growth were plated in 24 well plates at a plating density of 1×10^6^ parasites/mL in Schneider's medium supplemented with 20% FCS, pH 7.2. Drugs were diluted at the same medium and added to a parasite suspension in different concentrations, in triplicate. After 48 h of incubation, the parasites were counted and compared to the controls containing parasites in the absence of drugs. The drug concentration corresponding to 50% of the parasite growth inhibition was expressed as IC_50_. Three independent experiments were performed to confirm the results. Data are presented as mean and 95% CI.

### Intracellular amastigote assay

*L*. (*L*.) *amazonensis* and *L*. (*V*.) *braziliensis* promastigotes with 7–8 days and 5–6 days of growth, respectively, were harvested from cultures and added to a fresh Schneider's medium supplemented with 5% FCS, pH 6.0, and incubated at 32°C for 7 days for *L*. (*L*.) *amazonensis* and 4–5 days for *L*. (*V*.) *braziliensis* until a complete transformation to amastigote-*like*.

Peritoneal macrophages from BALB/c mice were harvested by washing with ice-cold RPMI 1640 medium, 4 days after induction with a 3% thioglycollate solution. Macrophages were diluted in RPMI 1640 medium (Sigma, Poole, United Kingdom) plus 10% FCS and plated in 24 well plates with a circular cover glass at a plating density of 2×10^5^ macrophages/well. Macrophages were allowed to adhere for 2 h at 37°C and 5% CO2, when the medium was replaced by a fresh one and incubated overnight.

Macrophages were infected with amastigote-*like* parasites from *L*. (*L*.) *amazonensis* or *L*. (*V*.) *braziliensis*. The parasites were counted in a Neubauer's chamber and adjusted to a macrophage-amastigote ratio of 1∶8. Infected cultures were maintained at 37°C and 5% CO_2_. After 4 h, extracellular parasites were removed by washing, and a fresh medium containing either TPM compounds, no drug, or the reference drug. AmB (Fungizone®-Bristol- Meyers Squibb Pharmaceutics Ltda, Bedford, USA) was the chosen reference drug and was used at 0.2 µg/mL. After 72 h, the cover glass was removed from the well, washed in RPMI 1640 medium, set in microscope blades, fixed with methanol, and stained with Giemsa for evaluation. Under the immersion microscope, infection indexes (number of amastigotes/100× percentage of infected macrophages) were determined by counting the numbers of intracellular amastigotes in 100 macrophages. The experiments were considered valid only when the control group (without drugs) displayed at least 80% of infection. Each point was tested in triplicate and three independent experiments were then performed. Results are presented as mean and 95% CI [25].

### Cytotoxicity

To evaluate the toxicity of selected compounds, an *in vitro* cytotoxicity assay on macrophages from BABL/c mice was performed through 3-(4,5-dimethyithiazol-2-yl)-2,5- diphenyltetrazolium bromide (MTT) (Sigma, Poole, United Kingdom) assay. Briefly, peritoneal macrophages from BALB/c mice were harvested, as described previously and plated in 96- well-flat-bottom microplates at a plating density of 1×10^5^ macrophages/well. Macrophages were allowed to adhere for 2 h at 37°C and 5% CO_2_, at which time the medium was replaced by a fresh one and incubated over night. Then, the cells were exposed to ten points of serial dilution of TPM 1, 2, 6, 9 or GV (0.195 and 20 µM), which were used to obtain a curve to determine the IC_50_. After 68 h of incubation, 10 µL of MTT (10 mg/mL) was added to each well and the plates were further incubated for 4 h. The enzymatic reaction was then stopped by addition of 100 µL of 50% isopropanol–10% sodium dodecyl sulfate solution. The optical density at 570 nm was quantified using an ELISA plate reader (BioSource, Inc., EUA). Three independent experiments, in triplicate, were performed to determine the cytotoxicity and data were expressed as mean and 95% CI. An experiment was also done to control the possibility of dye color interference on MTT assay. TPM 1, 2, 6, 9 and GV were plated diluted in RPMI for 68 h and then the MTT was added. The optical density at 570 nm was measured using an ELISA plate reader (BioSource, Inc., EUA).

### *In vivo* assay

#### Gel formulation

The formulation of the GV and TPM 6 gel was prepared by mixing equal amounts of a 2% hydroxyethylcellulose gel (HEC; Natrosol 250 HR, Aqualon) and a 2% GV or TPM 6 hydroethanolic solution (mixture ethanol/water 1/5), until a homogeneous preparation had been attained. Therefore, GV and TPM 6 concentration in these formulations was 1%. The gel lower concentrations for dose-response experiments was obtained by diluting the 1% GV gel with 1% HEC gel.

#### Treatment of infected animals

BALB/c mice (females, 5–6 weeks old) were inoculated with 1×10^7^ stationary growth phase promastigotes of *L (L.) amazonensis* through subcutaneous injections at the base of the tail, after trichotomy.

To evaluate the *in vivo* efficacy of GV and TPM 6, after development of ulcerated lesions (average diameter of 7 to 9 mm), BALB/c mice were divided into three groups. For treatment with TPM 6 and GV, lesions were covered with 50 µl of a gel formulation containing either 1% GV or 1% TPM 6, twice a day, for 20 days, using an Eppendorf pippetor. Control group: animals from control group were treated with the gel formulation without GV or TPM 6 (placebo). The treatment efficacy was evaluated through of the parasite quantification at the site of infection (see below).

Afterwards, a dose-effect study of GV was performed. BALB/c mice, presenting ulcerated lesions (average diameter of 7 to 9 mm), were divided into four groups, according to lesion size, to assure similar average lesion size among treated groups. The GV gel formulation was applied topically at 0.1, 0.5 or 1.0% twice a day, for 20 days. Control group: animals from control group were treated with the gel formulation without GV (placebo). The treatment efficacy was evaluated through of the parasite quantification at the site of infection (see below).

#### Parasite quantification

Three days after the interruption of treatment, the number of viable parasites at the site of infection was quantified by a limiting-dilution assay. Skin fragments from ulcerated lesions, were homogenized with a tissue grinder in Schneider's modified medium supplemented with 10% bovine fetal serum and 100 U/mL penicilin and 100 µg/mL streptomycin. Next, the tissue was centrifuged at 50 g for two minutes for sedimentation (Hitachi, Himac). The supernatant was separated and centrifuged again at 1700 g for 15 minutes (Express, Jouan). The pellet formed was resuspended in 1 mL of Schneider's modified medium supplemented with 10% FCS and 1% of a 100 U/mL penicillin and 100 µg/mL streptomycin solution. The homogenate was submitted to serial dilutions in duplicates in sterile 96 well culture plates and incubated at 23°C. Each well was examined for the presence of parasites, and the number of parasites was quantified by the highest dilution at which parasites could grow over a 7-day period. The lowest dilution that parasites were detected was 10^−1^, which was considered the limit of quantification.

### Statistical Analysis

The data were processed using MiniTab 15.1 and Sigma Stat 3.5 software. For *in vitro* assay, IC_50_ values were calculated by linear regression analysis. The statistical significance of differences among groups was evaluated using the one-way analysis of variance (ANOVA) test followed by Tukey's test. The Kruskal–Wallis non-parametric test followed by Dunn's Method for multiple comparisons was used to compare parasite quantification among groups. The difference was considered significant when the p value was less than 0.05.

## Results

### Promastigote assay

**Figure 2:**
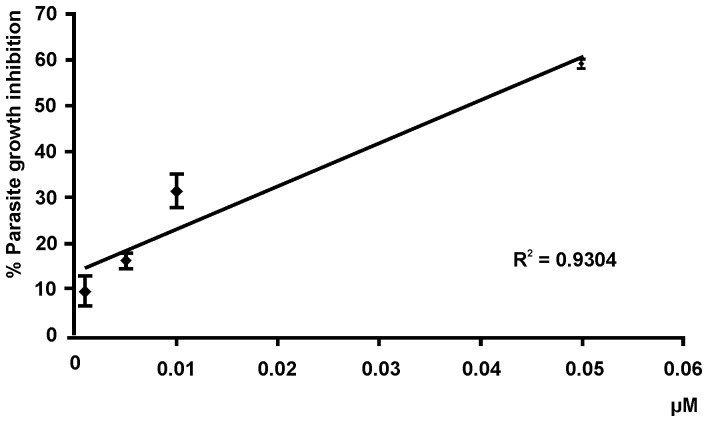
Dose-effect analysis of TPM 6 against *L (L.) amazonensis*. Promastigotes of *L*. (*L*.) *amazonensis* were plated in 24 well plates at a plating density of 1×10^6^ parasites/mL in Schneider's medium supplemented with 20% FCS, pH 7.2. TPM 6 was diluted in the same medium and added to parasites suspension at 0.001; 0.005; 0.01 and 0.05 µM, in triplicate. After 48 h, the parasites were counted and compared to the controls containing parasites in absence of drugs. Three independent experiments were done and the results were analyzed with MiniTab® Program. Data are the mean ± SD.

All ten TPM compounds were initially tested against *L. (L.) amazonensis* promastigotes. Figure 2 shows the results obtained for TPM 6. A linear relationship between the drug concentration and the parasite growth inhibition was obtained for TPM 1, TPM 2, TPM 6, TPM 9 and GV. Table 2 summarizes the data of IC_50_ obtained. The highest activity was observed for GV (IC_50_ 0.025 µM), followed by TPM 6, TPM 1, TPM 2 and TPM 9. For 5 out of 10 compounds evaluated (TPM 3, TPM 4, TPM 5, TPM 7 and TPM 10) the IC_50_ could not be precisely calculated as the compounds had a low activity against *L. (L.) amazonensis*, requiring higher concentrations, which exceeded the maximum EtOH concentration of 0.1% (data not shown). The compounds presenting the highest activity were then selected and tested on *L. (L.) major* and *L. (V.) braziliensis* promastigote assays. As observed in table 2, the IC_50_ determined for these compounds were similar for the three species tested, except for TPM 6, which showed a lower activity against *L. (V.) braziliensis* than that observed for *L. (L.) amazonensis*.

**Table 2:** *In vitro* anti-leishmanial activity of TPM compounds expressed as IC_50_ (µM) on promastigotes assay.

TPM	IC_50_ (µM)		
	*L.(L.) amazonensis*	*L.(L.) major*	*L.(V.) braziliensis*
TPM 1	0.436	0.567	0.492
	(0.375; 0.497)	(0.521; 0.613)	(0.445; 0.539)
TPM 2	0.546	0.764	0.551
	(0.462; 0.630)	(0.677; 0.851)	(0.476; 0.626)
TPM 3	>1.0	n.d.	n.d.
TPM 4	>2.0	n.d.	n.d.
TPM 5	>1.0	n.d.	n.d.
TPM 6	0.031^a^	0.045^a,b^	0.063^b^
	(0.026; 0.036)	(0.041; 0.049)	(0.054; 0.072)
TPM 7	>5.0	n.d.	n.d.
TPM 9	0.769	0.734	0.839
	(0.614; 0.924)	(0.658; 0.810)	(0.745; 0.933)
TPM 10	>4.0	n.d.	n.d.
GV	0.025	0.034	n.d.
	(0.016; 0.034)	(0.029; 0.039)	

IC_50_ values correspond to mean and 95% CI of results obtained from triplicates; n.d., not determined; data obtained for linear regression on MiniTab® 15.1 software, a,b p<0,05 compared IC_50_ determined for *L.(L.) amazonensis*, *L.(L.) major* and *L.(V.) braziliensis* .

### Intracellular amastigote assay

The compounds selected from the promastigote assays were subsequently tested on intracellular amastigote assays. Table 3 summarizes the results obtained. TPM 6 was the most effective compound against intracellular amastigotes of *L. (L.) amazonensis*, followed by GV, TPM 9, TPM 1 and TPM 2. Similar findings were observed for *L. (V.) braziliensis*, except for GV, which it was not tested against this species. The mean value of parasite growth inhibition observed with the control drug (0.2 µg/ml AmB) was 98% for *L. (V.) braziliensis* and 99.5% for *L. (L.) amazonensis*.

**Table 3:** Cytotoxicity, anti-leishmanial i*n vitro* activity and selectivity index (SI) of TPM 1, TPM 2, TPM 6, TPM 9 and GV against *L. (L.) amazonensis* and *L. (V.) braziliensis* on intracellular amastigotes assay.

TPM	Cytotoxicity	*L. (L.) amazonensis*		*L.(V.) braziliensis*	
	IC50 (µM)	IC_50_ (µM)	SI	IC_50_ (µM)	SI
TPM 1	8.21	0.76	10.80	0.52	15.78
	(7.46; 8.96)	(0.53; 0.99)		(0.23; 0.81)	
TPM 2	9.49	1.59	5.97	1.53	6.20
	(8.68; 10.30)	(1.25; 1.93)		(1.07; 1.99)	
TPM 6	4.16	0.10	41.60	0.10	41.60
	(3.18; 5.14)	(0.08; 0.11)		(0.09; 0.11)	
TPM 9	7.03	0.34	20.68	0.17	41.35
	(6.07; 7.99)	(0.29; 0.39)		(0.08; 0.26)	
GV	4.03	0.17	23.71	n.d.	n.d.
	(3.36; 4.70)	(0.16; 0,18)			

IC_50_ values correspond to mean and 95% CI of results obtained from triplicates; n.d., not determined; data obtained from linear regression on MiniTab® 15.1 software; mean value of parasite growth inhibition observed for control drug (0.2 µg/ml AmB) was 98% for *L. (V.) braziliensis* and 99.5% for *L. (L.) amazonensis*.

### Cytotoxicity

The MTT assay was performed to determine the cytotoxicity of TPM 1, TPM 2, TPM 6, TPM 9 and GV. Table 3 summarizes the results of cytotoxicity assays against peritoneal macrophages from BALB/c mice. The ratio of cytotoxicity to biological activity was used to determine the selectivity index (SI) of the compounds (Table 3). It is generally considered that biological efficacy is not due to *in vitro* cytotoxicity when this index is ≥10 [23]. The IC_50_ values observed in macrophage assays for TPM 1, TPM 2, and TPM 9 were higher than that observed for TPM 6 and GV, indicating that those compounds provide lower toxicity to macrophages. However, TPM 6 and GV presented higher selectivity indexes as compared to TPM 1, TPM 2 and TPM 9 for *L. (L.) amazonensis* infected macrophages. TPM 6 also presented the highest SI for *L. (V.) braziliensis* infected macrophages (Table 3).

### *In vivo* assay

**Figure 3:**
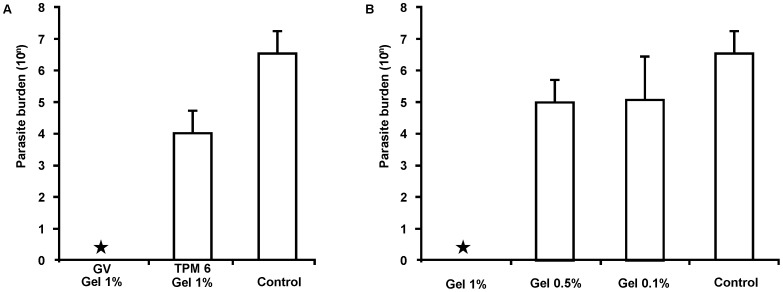
*In vivo* efficacy of GV and TPM6 topical treatment in *L (L.) amazonensis*-infected BALB/c mice. Female BALB/c mice were infected with *L (L.) amazonensis* at the base of the tail; 6 weeks after inoculation. **A**) Lesions were covered with 50 µl of a gel formulation containing either 1% GV or 1% TPM 6, twice a day, for 20 days. Animals from control group were treated with the gel formulation without GV or TPM 6 (placebo). The treatment efficacy was evaluated through of the parasite quantification at the site of infection. **B**) Dose-effect study of GV. The GV gel formulation was applied topically at 0.1, 0.5 or 1.0% twice a day, for 20 days. Animals from control group were treated with the gel formulation without GV (placebo). In both experiments, parasite numbers recovered from lesions were evaluated by a limiting dilution assay (* p<0.05 when compared to control group) (n = 5), three days after interruption of treatment. Two independent experiments were done and the results were analyzed with SigmaStat® Program.

The quantification of parasites within lesions was used to evaluate the efficacy of different treatments in BALB/c mice infected with *L. (L.) amazonensis*. First, the efficacy of GV and TPM 6 in a 1% gel was compared to a control group that received placebo. As seen in figure 3A, treatment with TPM 6 gel led to a significant decrease in the parasite burdens at site of infection, from 1×10^7^ (control group) to 1×10^4^ (TPM 6 treated group), whereas, no parasites were found at lesion site in the GV treated group.

In a dose-effect assay, GV was tested in a gel either at 0.1, 0.5 or 1%. Five animals per group were treated twice a day for 20 days, as above described. As shown in Figure 3B, the number of parasites within the lesion decreased when gel concentration were increased, although a linear dose-response has been not observed. The number of parasites in the control group (2.2×10^7^) was higher than that observed in the groups treated with GV gel at 0.1% (2.2×10^6^), 0.5% (2.62×10^5^), or 1% (parasites were not detected). Statistical analysis showed a significant reduction in parasite numbers only in 1% GV treated group when compared with the control group (p<0.05).

## Discussion

Given the worldwide prevalence of *Leishmania* infection in countries that have low budgets for health care, finding a safe and inexpensive treatment for leishmaniasis is still an unmet need. In this study, 10 novel TPM were evaluated against promastigotes and amastigotes from 3 species of *Leishmania*, recognized worldwide as major etiological agents of CL. The most effective compounds proved to be GV and TPM 6 for all the *Leishmania* species tested. Overall, there was no significant difference in the efficacy of the same compound against the promastigotes of three different species of *Leishmania*.

Tests against intracellular amastigotes are more relevant to infer the sensitivity of anti- leishmanial drugs, since this is the parasite stage found in the vertebrate host [26]. In the present study, no significant differences were observed regarding the sensitivity of the different *Leishmania* species to each tested compound. In intracellular amastigote assays, the TPM compounds presented an IC_50_ between 0.10 µM and 1.59 µM for *L. (L.) amazonensis* and between 0.10 µM and 1.53 µM for *L. (V.) braziliensis*. Morais-Teixeira *et al*. [27], using the same methodology for intracellular amastigote assays described for Glucantime™, the first choice drug in CL treatment, an IC_50_ of 22.9 µg/mL (188.07 µM) for *L. (L.) amazonensis* and 24.2 µg/mL (198.75 µM) for *L. (V.) braziliensis*. Thus, the TPM compounds were between 10–100 fold more potent against *Leishmania* than the current standard of care. Moreover, based on the cytotoxicity data, the selectivity index could also be estimated. It is generally considered that biological efficacy is not due to *in vitro* cytotoxicity, when this index is ≥10 [28], as it was observed for TPM 1, TPM 6, TPM 9 and GV against *L. (L.) amazonensis* and TPM 1, TPM 6 and TPM 9 against *L. (V.) braziliensis*.

Two mechanisms of action have been ascribed to TPM. An initial study from our laboratory has shown that TPM are potent inhibitors of NADPH oxidase [29]. A second study of ours demonstrated that TPM, but not other NADPH oxidases, also localize in mitochondria and form covalent adducts with thioredoxin 2 (Trx2) [30]. We believe that the first mechanism can be excluded for direct activity against *Leishmania*, as compounds that were inactive against *Leishmania* species show potent NADPH oxidase inhibitory activity, namely the compounds with larger aromatic substituent. The possible role of Trx2 as a target for *Leishmania* is particularly attractive given a recent report of the role of Trx2 in malaria parasite survival, and may provide an explanation of the activity of TPM against both promastigotes and amastigotes of *Leishmania* [31].

Among the TPM compounds, TPM6, TPM 9 and GV demonstrated the highest selectivity indexes (Table 3). In agreement to the findings observed on the *in vitro* assays, both TPM 6 and GV showed high activity against parasites *in vivo*. TPM 6 decreased the parasite burdens by three orders of magnitude, while the 1% GV gel promoted complete elimination of parasites in treated animals. In a dose-response experiment with GV gel, a linear dose dependent response was not observed, but again complete elimination of parasite burden was observed in animals treated with the GV gel at 1%. Similar results were observed in preliminary experiments in *L. (V.) braziliensis* infected hamsters (data not shown).

Limitations of this study may include the use of an extreme susceptible experimental model and the lack of an extended period to follow up lesions healing. It is noteworthy that previous data have indicated that intramuscular administration of Glucantime, at doses equivalent to those used in human chemotherapy, to either hamsters infected *L. (V.) braziliensis* or BALB/c mice infected with *L. (L.) amazonensis* did not lead to significant reductions in lesions size [15]. Similar findings were observed when testing topical administration of PA to BALB/c mice infected with either *L. (L.) amazonensis* or *L. (V.) braziliensis* [15], [32], [33].

Thus, for the first time, we have demonstrated that GV and other members of the TPM class are effective against *Leishmania* species *in vitro* and *in vivo*. Humans have a long history of topical GV use as an antibacterial and antifungal agent, and we extend the potential use of GV as a potent antileishmanial agent. In addition, humans have tolerated systemic exposure of GV as well, given that GV is routinely added to transfused blood in areas where Chagas disease is prevalent [19]–[24].

This study indicates that some TPM derivatives have *in vitro* anti-leishmanial activity and that this activity is not limited to a single species. In addition, we have demonstrated that topical GV is highly effective against the usually refractory species *L. (L.) amazonensis in vivo*, perhaps making it an alternative treatment agent where species diagnosis is not possible. The findings described herein are of public health relevance for the following reasons. First, these drugs are inexpensive and stable at room temperature, making them ideal for use in areas where *Leishmania* is endemic. Second, GV is readily accessible and has an established safety record, making clinical trials rapidly feasible. Finally, gentian violet has anti-angiogenic properties, which might lead to an enhanced host response, in addition to direct anti-parasitic activity.
